# The influence of ambient environmental factors on breakthrough cancer pain: insights from remote health home monitoring and a proposed data analytic approach

**DOI:** 10.1186/s12904-024-01392-9

**Published:** 2024-03-02

**Authors:** Nutta Homdee, John Lach, Leslie Blackhall, Virginia LeBaron

**Affiliations:** 1 Center for Research Innovation and Biomedical Informatics, Faculty of Medical Technology, Mahidol University, Nakhon Pathom, Thailand; 2 The George Washington University School of Engineering & Applied Science Science & Engineering Hall, Washington, DC USA; 3 University of Virginia School of Medicine, Charlottesville, VA USA; 4 University of Virginia School of Nursing, Charlottesville, VA USA

**Keywords:** cancer, Pain management, Oncology, Environmental factors, Remote health monitoring

## Abstract

**Background:**

Breakthrough cancer pain (BTCP) is primarily managed at home and can stem from physical exertion and emotional distress triggers. Beyond these triggers, the impact of ambient environment on pain occurrence and intensity has not been investigated. This study explores the impact of environmental factors on the frequency and severity of breakthrough cancer pain (BTCP) in the home context from the perspective of patients with advanced cancer and their primary family caregiver.

**Methods:**

A health monitoring system was deployed in the homes of patient and family caregiver dyads to collect self-reported pain events and contextual environmental data (light, temperature, humidity, barometric pressure, ambient noise.) Correlation analysis examined the relationship between environmental factors with: 1) individually reported pain episodes and 2) overall pain trends in a 24-hour time window. Machine learning models were developed to explore how environmental factors may predict BTCP episodes.

**Results:**

Variability in correlation strength between environmental variables and pain reports among dyads was found. Light and noise show moderate association (r = 0.50–0.70) in 66% of total deployments. The strongest correlation for individual pain events involved barometric pressure (r = 0.90); for pain trends over 24-hours the strongest correlations involved humidity (r = 0.84) and barometric pressure (r = 0.83). Machine learning achieved 70% BTCP prediction accuracy.

**Conclusion:**

Our study provides insights into the role of ambient environmental factors in BTCP and offers novel opportunities to inform personalized pain management strategies, remotely support patients and their caregivers in self-symptom management. This research provides preliminary evidence of the impact of ambient environmental factors on BTCP in the home setting. We utilized real-world data and correlation analysis to provide an understanding of the relationship between environmental factors and cancer pain which may be helpful to others engaged in similar work.

**Supplementary Information:**

The online version contains supplementary material available at 10.1186/s12904-024-01392-9.

## Background

Over 90% of advanced cancer patients experience pain, and about 50 to 75% of cancer patients with pain suffer from sudden, intense breakthrough cancer pain (BTCP), which can occur despite using pain medications [1–3]. BTCP significantly affects the quality of life for patients and their caregivers looking to manage their pain at home [3–5]. Thus, preventing and managing BTCP is crucial in cancer pain care [6–8].

While triggers like physical exertion and emotional distress are known, the impact of home environmental factors (such as light, temperature, humidity, noise) on BTCP remains understudied [9–12]. Multiple investigations indicate that environmental conditions in hospitals may affect patients’ health outcomes [11, 12]. A comprehensive review revealed that factors such as music, natural light levels, and artificial ambient lighting can influence the pain experienced by hospitalized patients with diverse diagnoses/illnesses [10]. However, there is limited research regarding how environmental factors within the home environment can affect cancer pain. We aim to fill this gap by investigating the influence of environmental factors on cancer pain in the home setting, where cancer pain is most commonly addressed and managed [7].

### Overview of the BESI-C system

Our interdisciplinary team previously developed the BESI-C (Behavioral and Environmental Sensing and Intervention system for Cancer) remote health monitoring system, which utilizes smartwatches and ambient sensors to collect data on home environmental factors, as well as behavioral and physiological data from patients and their primary family caregiver. BESI-C includes a custom smartwatch application that allows both patients and caregivers to self-report BTCP events, record medication intake and efficacy, as well as quality of life variables such as sleep quality and quantity, physical activity, and psychological distress. Our user-centered design process [13], overall study protocol [14], and results from initial feasibility and acceptability testing of BESI-C [15] have been previously reported [16].

**Fig. 1:**
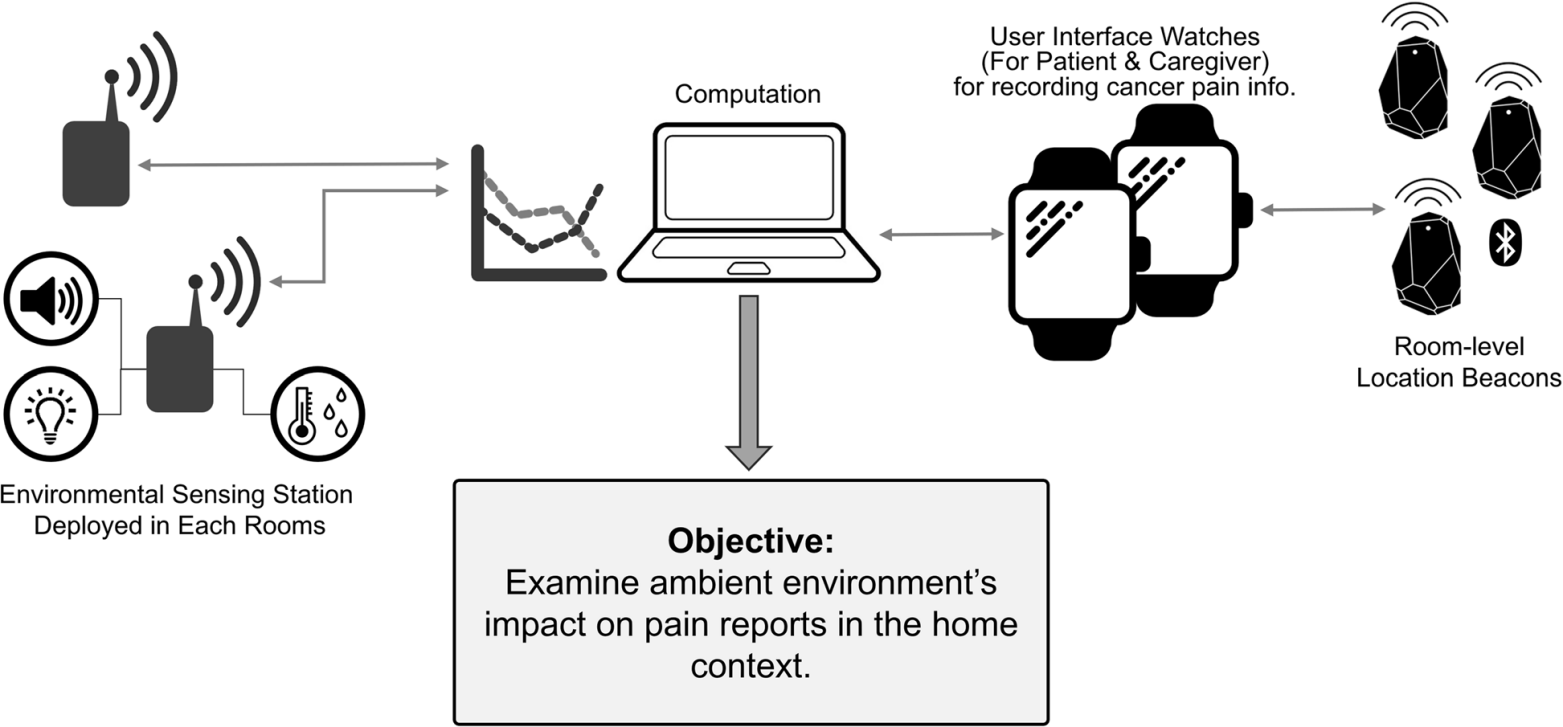
Overview of the BESI-C remote health monitoring system. Ambient environmental data are collected via environmental sensing stations. Smartwatches are used to collect physiological data from the patient and the caregiver, record pain events and complete Ecological Momentary Assessments (EMAs), and locate users in the home

This paper focuses on a unique aspect of the BESI-C system: environmental sensors collecting anonymized data on light, ambient noise, barometric pressure, temperature, and humidity in the home and how these data correlate with self-reported pain and distress data from patients and family caregivers. The selection of these environmental variables is based on literature highlighting their potential impact on the quality of life for palliative care patients [17]; validation by previous conducted dyad interviews [13]; and technological feasibility [16, 18, 19]. Figure 1 shows an overview of the BESI-C system architecture used for the collection of the data presented in this paper.

Analyzing data from BESI-C using statistical methods and machine learning helps uncover trends in BTCP-related data and potential environmental influences. For instance, statistical analysis can reveal correlations between light/temperature and BTCP frequency/intensity. Machine learning, including decision trees and neural networks, is effective in identifying complex patterns and relationships in data related to symptoms and environmental factors [20]. These algorithms are especially valuable for uncovering intricate connections not easily discernible through traditional statistical analysis [21].

The primary objective of this analysis and paper is to investigate the hypothesis that ambient environmental factors impact the frequency and severity of BTCP in home settings, offering insights for enhanced pain management in cancer patients. Our contributions include: 1) Analyzing real-world data for correlations between environmental factors and BTCP; 2) Exploring how the ambient environment influences individual BTCP instances and overall frequency/severity within a 24 hour period; 3) Extracting features from real-time environmental data to develop machine learning models to predict episodes of BTCP experienced in the home setting and 4) Comparing patient-reported pain episodes with caregiver-reported observations and their connection to the ambient environment. Importantly, this study provides insights into the development of personalized cancer pain interventions at home, potentially informing real-time environmental modifications to alleviate pain and distress for patients and caregivers.

## Methods

### Data acquisition

This paper reports findings from five BESI-C deployments (*n* = 5) in patient and caregiver homes in Central Virginia from April 2019 to December 2019. The BESI-C system collected self-reported pain events and contextual environmental data for approximately 2 weeks (9 to 15 days per deployment). The analysis integrates environmental data from BESI-C sensors in participant homes with pain events reported via smartwatches by both patients and caregivers. The focus is on the frequency and severity of breakthrough cancer pain (BTCP) events, assessed through user-initiated Ecological Momentary Assessments (EMAs) (brief surveys) on smartwatches. BTCP events are defined when a pain event is marked and an EMA is completed on the smartwatch, as detailed in previous reports [15]; of note, patients are asked to complete an EMA when they believe they are experiencing cancer-related pain; caregivers are asked to complete an EMA when they believe the patient is experiencing pain. Severity of pain events was assessed by the Numeric Rating Scale (0 = no pain to 10 = worst pain) [22, 23]; frequency of pain events was assessed by the time stamps of a completed pain report EMA. Consistent with the guidelines of our healthcare institution, a reported pain level of 5 or higher was considered a high-severity pain event.

**Fig. 2:**
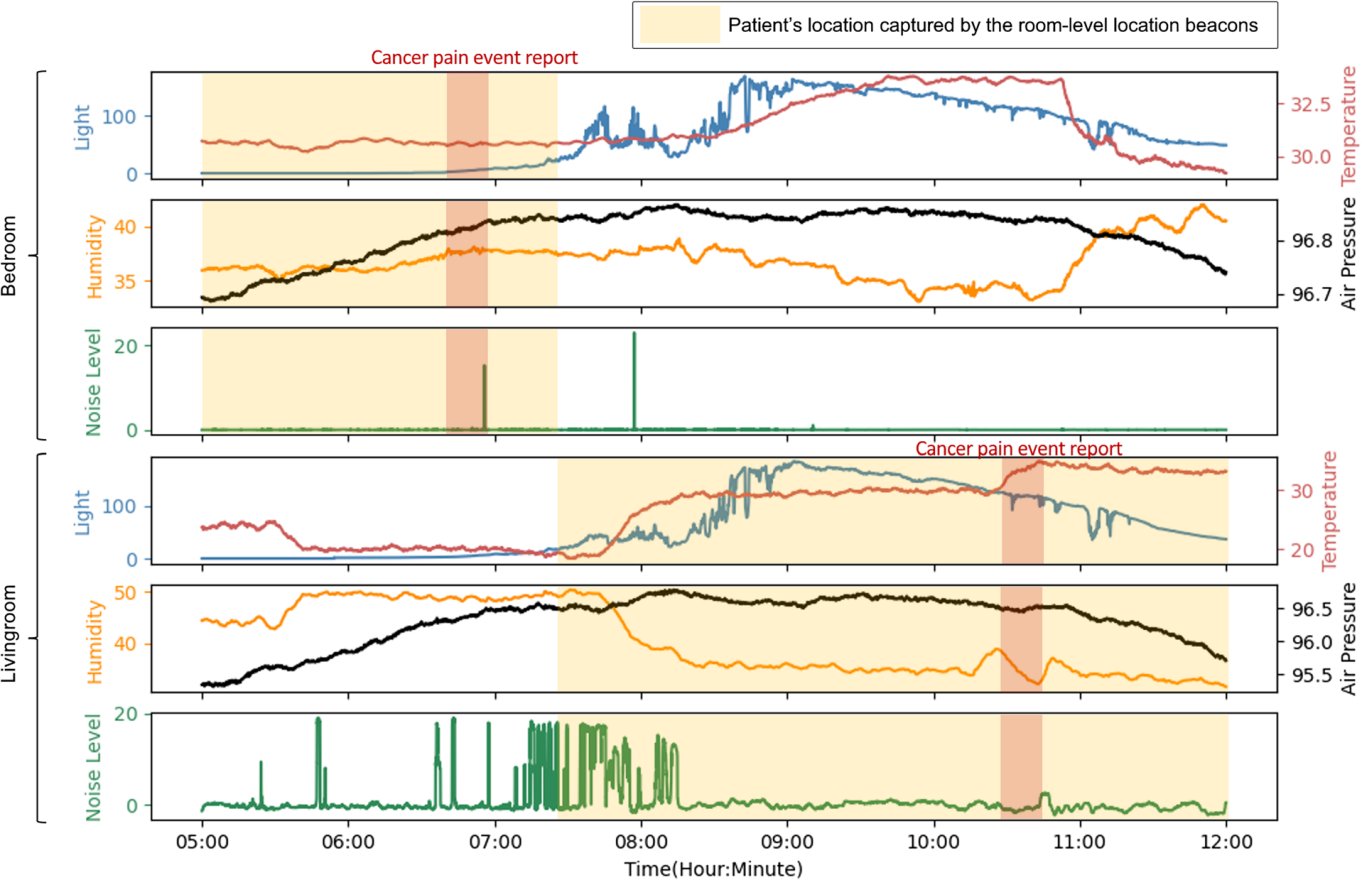
Example of environmental data with BTCP reports from a BESI-C deployment. The two red-highlighted areas represent two self-reported pain events, one in the bedroom, another in the living room. The yellow-shaded areas represent the patient’s location estimated by Bluetooth beacons. *NOTE: The collected environmental data and their units are light level (Lux), temperature (°C), humidity (%RH), barometric pressure (kPa), and noise level (dB)*

In addition to enabling participants to record pain events, smartwatches tracked participants’ locations using Bluetooth beacons strategically placed in selected rooms of their homes (note: placement of sensors within the home was always determined in partnership with the dyad and excluded sensitive areas, such as bathrooms).. This location information was used to correlate reported pain events with the most relevant room-level ambient environmental sensor data based on Bluetooth signal strength. For example, if the Bluetooth beacon revealed that a patient recorded a pain event closest to the kitchen, the data from the kitchen environmental sensor were used for correlation analysis. Figure 2 illustrates the collected data from environmental sensors, patient location, and self-reported BTCP events.

### Data pre-processing and environmental features

We utilized Python programming to apply data pre-processing techniques, including the removal of sensor interference signals (unwanted electrical signals from sensor hardware) and extraction of relevant features [24]. In our exploratory analysis, lacking a published standard, we chose a 5-second filter size with the rationale that a 5-second window size is a balance between interference reduction and preserving relevant signal. Subsequently, we employed data segmentation to divide environmental data into chunks, aiming to reduce complexity and facilitate feature extraction capturing temporal patterns. Using a sliding window technique, we created overlapping segments of fixed length from continuous data streams [25]. For analyzing environmental impacts on pain events, we segmented environmental data preceding each pain report into variable window sizes (5-minute to 60-minutes). Next, we employed feature extraction to convert raw sensor data in each segment into more informative representations, reducing redundancy and irrelevance. Table 1 summarizes the statistical features used to study the relationships between environmental factors and BTCP reports.

**Table 1:** Summary of extracted features and their relevance to ambient environmental data segments

Extracted Feature Category	Extracted Feature Name	Description/Rationale
Measures of central tendency	Mean	Measures of central tendency describe the center or typical value of a dataset. The mean is the average value of the data which helps us capture the general level of the data and provide information about the baseline behavior.
Measures of dispersion	Standard deviation (SD)	Measures of dispersion describe how spread out the data are. The SD measures how much the data varies from the average value.
	Minimum (Min) & Maximum (Max)	Min and Max values give the lower and upper range of the data which can be beneficial when certain ranges of values are indicative of specific conditions (e.g., pain severity/intensity)
	Median deviation (MD)	The median deviation is a measure of how much the data varies from the middle value [26]. We extract median deviation (MD) related features which consist of mean-MD, max-MD, and min-MD so we can capture variability while being less influenced by extreme values (e.g., high light levels at night show high MD feature, but low Mean compared to daytime).
Measures of shape	Slope	Slope measures the shape (or pattern) of the data distribution by measuring how steeply a line fits to the data. This feature is important for capturing trends, identifying periods of growth or decline.
	Mean-crossing-rate (MCR)	The MCR measures how often the data goes above and below its average value [27]. This feature is useful for characterizing oscillatory behavior and cyclical patterns in the data.

### Data analysis

#### Correlation analysis between ambient environmental factors and BTCP

Two correlation analysis approaches were utilized with different unit of analysis to investigate the relationship between ambient environment and BTCP frequency and severity. In the first correlation analysis, the individual BTCP correlation analysis, we looked at each individual BTCP event and environmental features prior to the BTCP event. For example, with a 15-minute window, if a patient reported BTCP at 2:00 PM, environmental features between 1:45 PM and 2:00 PM were considered for the point-biserial correlation. The unit of analysis was the individual BTCP event. In the second correlation analysis, daily (24 hr) breakthrough pain events correlation, we looked at the frequency/intensity of BTCP in a 24-hour period and environmental features in the same 24-hour period. For example, if a patient reported five BTCP episodes on a day with an average light level of 1000 lx, compared to another day with an average light level of 400 lx and 2 BTCP reports, the correlation between average environmental values and the number of BTCP episodes would be analyzed using Pearson correlation. The unit of analysis is each day (24-hour period) of the deployment.

### Individual comparison of environmental features and breakthrough Cancer pain episodes

The first approach examined the impact of the ambient environment on individual BTCP events by calculating Point-biserial correlation coefficients between environmental features (i.e., light level, temperature fluctuation, and noise level) and the occurrence of BTCP episodes reported by patients and caregivers [28]. These coefficients measured the relationship between continuous environmental variables and the binary occurrence of individual BTCP events. Environmental features were segmented at variable window sizes (5 to 60 minutes) preceding each pain report. To compare environmental conditions during periods with and without pain events, ‘control’ periods without pain reports were randomly selected using a Python program, excluding those within 1 hour of pain report timestamps. Then, Pearson correlation coefficients [29] were computed to evaluate the relationship between ambient environment and reported pain severity (patient’s self-report or caregiver’s observed level). For instance, with a 15-minute window, if a patient reported BTCP at 2:00 PM, environmental features matching the patient’s location between 1:45 PM and 2:00 PM were considered, and Pearson correlation coefficients were calculated with the reported pain intensity using the Numeric Rating Scale (NRS).

### Daily comparison of environmental factors and breakthrough Cancer pain episodes

The second correlation approach examined the daily impact of ambient environmental factors on the frequency and intensity of BTCP. This involved analyzing the correlation between the number and average severity of BTCP episodes reported in a 24-hour period by patients and caregivers and the 24-hour average values of environmental factors (light level, temperature, humidity, barometric pressure, and noise level). Daily average values for environmental factors were calculated over the data collection period (9–15 days, depending on deployment duration). For instance, if a patient reported five BTCP episodes on a day with an average light level of 1000 lx, compared to another day with an average light level of 400 lx and 2 BTCP reports, the correlation between average environmental values and the number of BTCP episodes would be analyzed using Pearson correlation.

#### Breakthrough Cancer pain prediction

In addition to correlation analysis, machine learning techniques predicted breakthrough cancer pain (BTCP) based on environmental factors (5-minute to 60-minute feature window sizes before pain events). Time-of-day was also considered as an input feature, with cyclical feature encoding used to address numerical representation challenges (e.g., 23:58 and 00:02). This method converts time-of-day into corresponding sine and cosine values [30, 31]. Before building BTCP prediction models, data normalization scaled sensor data to a common range, aiding to mitigate the influence of different units and scales of measurement on data analysis [32]. Min-max normalization was employed, rescaling the data to a range between 0 and 1.

Machine learning models, including naive Bayes, decision tree, random forest, support vector machine (SVM), and neural network, were trained to predict BTCP based on the environmental features [33, 34]. The models underwent training on 80% of the data using the 5-folds cross-validation approach [35], ensuring generalizability and minimizing overfitting. To address potential individual differences in environmental sensitivity, a personalized machine learning model was created for each unique dyad, allowing for personalized predictions. The remaining 20% of the data served as an independent testing dataset to evaluate model performance, ensuring that the model generalized well to new data [36].

Model performance was assessed using accuracy, sensitivity, specificity, area under the receiver operating characteristic curve (AUC), and Matthew’s correlation coefficient (MCC). Given dataset imbalance, MCC was particularly valuable, as is common in medical data [37]. The performance of the pain prediction models was compared, and the best-performing model and feature window sizes were selected for further analysis and discussion. Hyperparameters were tuned using a grid search approach, defining a search space as a grid to find optimal values for the prediction model [38, 39].

## Results

This section presents the results of BTCP correlations with environmental features, and the results of machine learning model performance for predicting BTCP. Below, Table 2 summarizes the result of the correlation analysis and the associated environmental features. The overall sample size for the correlation analysis was 566 events, including 283 self- reported BTCP events from patients and caregivers over the total five deployments and 283 ‘control’ periods without pain events. Here, we considered the correlation coefficient as low correlation when the value is below ±0.30, moderate correlation when the value lies between ±0.30 and ± 0.50, and high correlation when the value is above ±0.50 [40].

**Table 2:** Summary of environmental features that show the strongest association with patient BTCP of each deployment when compared with individual BTCP episodes and BTCP frequency/severity over 24 hours

Deployment Number (D.#)	Environmental feature associated with individual episodes of patient BTCP		Environmental feature associated with episodes of patient BTCP over a 24-hour time window	
	individual BTCP	individual BTCP severity	BTCP frequency	average BTCP severity
D.1 (*n* = 98)	Light MCR (r = −0.33)	Light Max (r = 0.26)	Humidity MD (r = 0.84)	Light MD (r = 0.57)
D.2 (*n* = 84)	Light Median Deviation (r = − 0.24)	Noise Median Deviation (r = 0.36)	Noise Max (r = 0.78)	Noise Mean & SD (r = 0.74)
D.3 (*n* = 48)	Light Minimum (r = 0.38)	Noise Mean (− 0.73)	Noise SD (r = 0.60)	Barometric pressure MCR (r = 0.83)
D.4 (*n* = 60)	Barometric pressure MD (r = 0.45)	Light MCR (r = −0.31)	Barometric pressure MD (r = 0.84)	Temperature MD (r = −0.82)
D.5 (*n* = 106)	Barometric pressure SD (r = 0.19)	Noise Median Deviation (r = 0.33)	Barometric pressure Max (r = 0.66)	Light MD (r = −0.59)

*r correlation coefficient*: *n number of patient BTCP and ‘control’ events.*

### Correlation analysis between environmental features and individual pain episodes

**Fig. 3:**
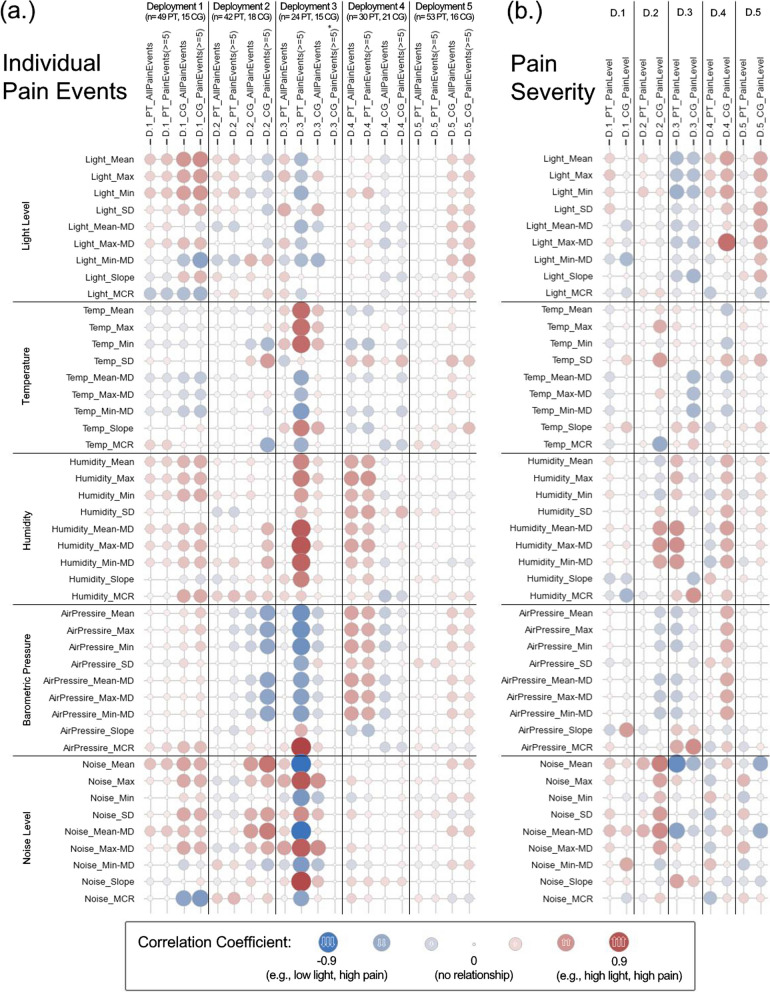
Correlation coefficient analysis results between environmental features and BTCP frequency and severities, per patient, caregiver, and dyad, reported by the patients and caregivers. Correlation methods used are Point-biserial and Pearson’s correlation for pain occurrence and severity, respectively. *In deployment 3, CG did not report any observed pain event with severity level more than or equal to 5. Note: n = number of pain reports; PT = patient; CG = caregiver; D = deployment; SD = standard deviation; MD = Median Deviation; MCR = Mean-Crossing-Rate

For the correlation analysis of individual pain events, environmental features preceding each pain report were compared with the frequency and severity of the pain reports. The 15-minute feature window is used for this analysis (see Appendix A for more details.) Pearson correlation coefficients between the environmental features and pain frequency and severity (per patient, caregiver, and deployment) are summarized in Fig. 3. In Fig. 3, the size of the circles represents the magnitude of the correlation coefficient, with larger circles indicating a higher correlation. The color of the circles indicates the direction of the correlation, with red representing a direct correlation (e.g., high light, high pain) and blue representing an inverse correlation (e.g., low light, high pain). Environmental factors analyzed include light level, temperature, humidity, barometric pressure, and noise level. Features extracted from each environmental factor include mean, max, min, standard deviation, median deviation features, slope, and mean-crossing-rate. The investigation into BTCP events using the biserial correlation coefficient showed associations with various environmental features, particularly light levels and ambient noise levels, across different deployments.

### Light levels and ambient noise

In Fig. 3, analysis of deployments 1 and 5 suggest that individual BTCP events were positively associated with high light levels, elevated noise levels, and noise fluctuations (as shown by high standard deviations). Similarly, the correlation analysis in deployment 3 showed associations between individual pain events and ambient noise related features. The result of pain severity correlations, conducted using Pearson’s correlation, showed similar trends in deployments 1 and 5. Both deployments exhibited moderate correlations between pain severity and environmental features, specifically light levels and ambient noise levels. For instance, in deployment 5, pain severity reports exhibited a correlation coefficient of 0.41 with mean light level, standard deviation, and median deviation of light. In deployment 3, assessments of pain severity from both patients and caregivers revealed moderate inverse correlations with all light level features, ranging from approximately 0.35 to − 0.47. The feature with the highest correlation was the maximum median deviation of the light level during deployment 4 and the caregiver’s pain severity observation, with a correlation coefficient of 0.71.

### Temperature and barometric pressure

Deployments 3 and 4 showed associations between temperature, barometric pressure, and pain events. The highest correlation coefficient observed during the individual pain events correlation analysis was in deployment 3 for the patient with high-severity pain reports (pain level ≥ 5/10), where the barometric pressure (MCR) showed a correlation coefficient of 0.90.

#### Correlation analysis between daily environmental features and daily BTCP episodes

**Fig. 4:**
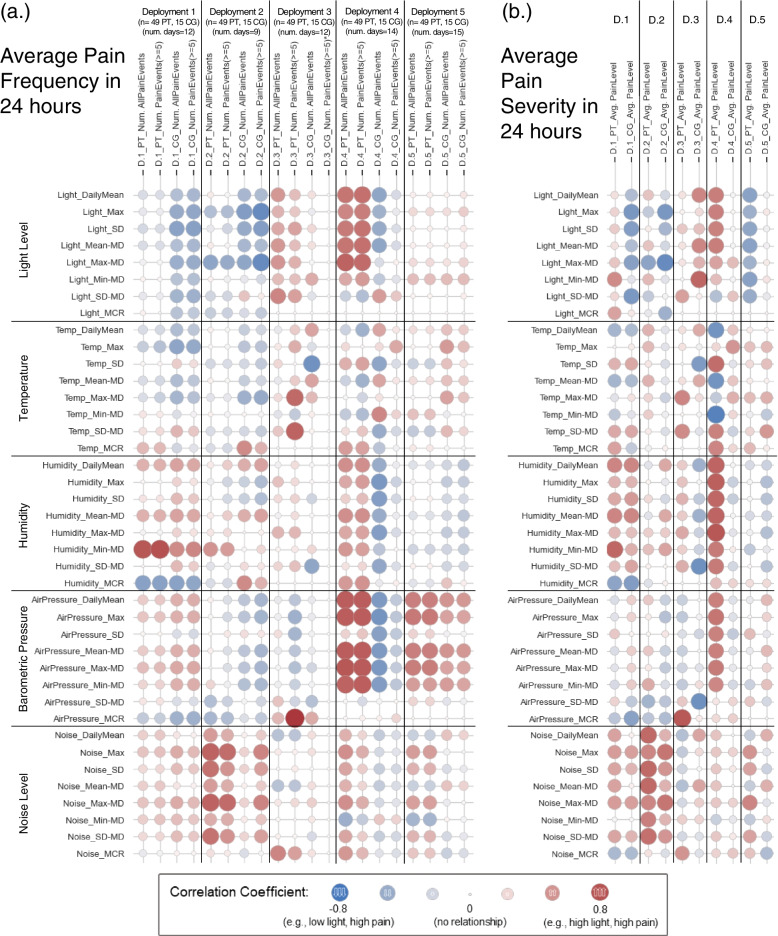
Correlation coefficients between ambient environment features and (a) average pain frequency (number of reported pain events) in 24 hours period, and (b) average pain severity in 24 hours period. *In deployment 3, CG did not report any observed pain event with severity level more than or equal to 5. Note: n = number of pain reports; PT = patient; CG = caregiver; D = deployment; SD = standard deviation; MD = Median Deviation; MCR = Mean-Crossing-Rate

The second correlation analysis approach aimed to investigate the overall impact of the ambient environment on the occurrence of BTCP by analyzing the correlation between the daily average values of environmental features and the daily average number and severity of BTCP episodes reported by patients and caregivers. This approach used Pearson’s correlation method for both the number and average severity of BTCP episodes reported over a 24-hour period. Fig. 4 illustrates the correlation coefficient between daily ambient environmental values (the 24-hour average values of environmental features) and daily average number and average severity of BTCP reported by patients and caregivers in a 24-hour period. Similar to Fig. 3, the circle size represents correlation strength (bigger circles indicate higher correlation coefficient), while the color indicates direction: red for direct and blue for inverse.

### Light and ambient noise

In Fig. 4, results from deployments 1, 2, and 4 suggest the influence of light features on BTCP. In deployment 1, light features showed an inverse correlation with patient’s pain severity and pain events as reported by caregiver, both displaying a coefficient of − 0.55 with the light’s SD (representing high fluctuation of light level in a day). Similarly, deployment 4 showed that patient pain reports displayed high correlation coefficients with light features, specifically daily light level, max light level, light SD, and light median deviation. However, in this deployment, both light features and barometric pressure features demonstrated an inverse correlation with caregiver pain reports. Deployment 4 also showed the correlation between pain severity and light level features (daily Mean, Max, SD, and Mean-MD). These features exhibited high direct correlations with the number of patient pain reports, while inversely correlating with the number of caregiver reports. Moreover, light level features displayed a significant correlation with the average patient-reported pain severity, featuring correlation coefficients of 0.62. Finally, in deployment 5, daily light levels demonstrated an inverse correlation with average pain severity.

### Humidity

In deployment 1, lower-than-normal humidity (Humidity Min-MD feature) was notably correlated with both the number of patient pain reports (all pain levels) and the number of high pain reports (severity level ≥ 5), with correlation coefficients of 0.84 and 0.87, respectively.

### Barometric pressure

In deployment 3, the MCR of barometric pressure exhibited a high correlation coefficient of 0.83 with the number of pain reports from patients experiencing pain levels ≥5. Deployment 4 demonstrated high direct correlations between light level features and barometric pressure features and the number of patient pain reports, while also revealing an inverse correlation with the number of caregiver reports. In deployment 5, barometric pressure features displayed direct correlations with both patient and caregiver number of pain reports.

### Machine learning for breakthrough Cancer pain prediction

This section demonstrates the use of machine learning to predict BTCP based on environmental features and time-of-day. A total of 594 data points were analyzed. The predictive models use a dataset that includes environmental data from 15 minutes (see Appendix A) prior to pain reports. The hyperparameters computed by the grid search method for machine learning models are reported in Appendix B [41].

**Fig. 5:**
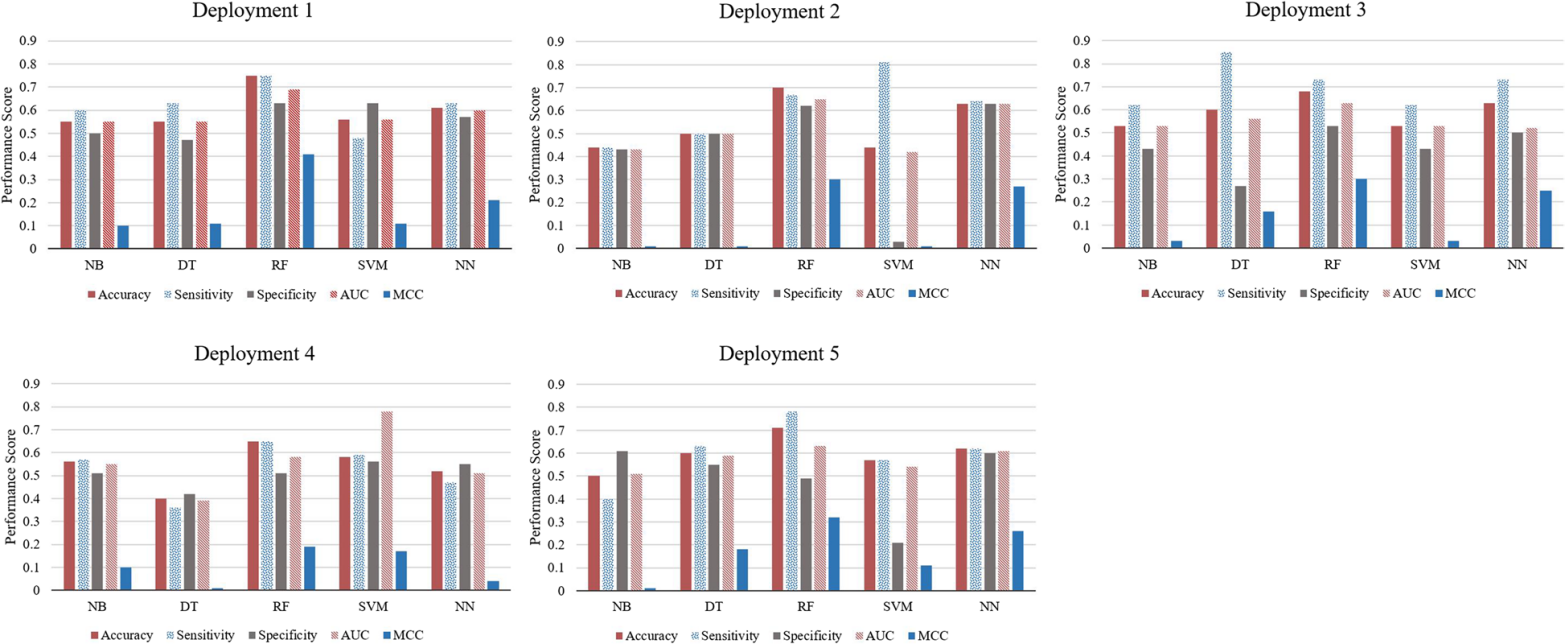
Comparison of performance of different machine learning models to predict BTCP events using 5-folds cross-validation on 80% of the dataset (475 out of the total 594 observations) is shown. Each deployment was trained and validated on their own data. *Note: NB = Naïve Bayes; DT = Decision Tree; RF = Random Forest; SVM = Support Vector Machine; NN = Neural Network; AUC = Area Under Curve; MCC = Matthew’s correlation coefficient*

For all deployments, our results in Fig. 5 showed that the random forest (RF) model was the best performing machine learning model, followed by the neural network model. The average accuracy from all deployments for predicting BTCP was 70%. When looking at the MCC, deployment 1 demonstrated the best performance using the RF model with an accuracy of 75%, sensitivity of 76%, specificity of 63%, AUC of 69%, and MCC of 0.4. Deployment 4 showed the least favorable performance, with the RF accuracy of 65%, sensitivity of 65%, specificity of 51%, AUC of 58%, and MCC of 0.19. The results of the individual correlation and pain event prediction, using the same 15-minute time window of environmental data prior to pain reports, are also consistent; showing the highest and lowest correlation coefficients between pain occurrences and environmental features in deployment 1 and deployment 4, respectively.

## Discussion

The results of this study suggest that environmental factors may have an impact on the experience of BTCP in the home setting. Both the correlation analysis and the pain event prediction models revealed associations between certain environmental features and the frequency and severity of BTCP events. These findings represent a significant contribution to cancer pain and remote sensing literature, as there has been little research to date regarding how to assess the impact of environmental variables on the experience of cancer pain in the home context. A better understanding of the role of environmental variables on cancer pain provides opportunities to recommend personalized, low-burden environmental modifications that could mitigate pain and distress for both patients and their family caregivers.

Both individual and broader (daily 24-trends) correlation analyses are important for understanding the relationship between environmental factors and BTCP. The individual analysis, focusing on the short-term (15-minute) environmental impact before a pain event, revealed strong correlations (0.70 to 0.90) between pain frequency and environmental data, though correlations with pain severity were lower (up to − 0.73). In other words, we found environmental factors more strongly influenced *how often* people experience BTCP events, but not necessarily the *perceived severity* of these events. Broader daily analyses, comparing average environmental values with pain frequency and severity within a 24-hour period, revealed even stronger associations (0.70 to 0.85 for frequency, 0.74 to 0.83 for severity). This suggests that considering daily trends, rather than isolated, individual pain reports, is likely to be more helpful in evaluating the true impact of environmental factors on BTCP. Machine learning models, particularly RF, effectively predicted BTCP based on environmental features and time-of-day, achieving approximately 70% accuracy across five deployments. This indicates the potential for building predictive models for in-home pain events based on ambient environmental factors.

Another interesting observation is the high correlation between light and noise features, such as mean light/noise level and max light/noise level, and BTCP across most deployment dyads. While this suggests that ambient light and noise may influence BTCP at home (e.g., heightened pain with increased light levels), an alternative explanation could be that pain is more prevalent during the day when individuals are active, walking around, and exerting themselves. To account for this, our analysis incorporates Mean Deviation features, such as the light MD feature, which accommodates the typically low light levels during nighttime. Thus, if we find a high correlation between light levels and pain, but not with light MD, we can infer that daytime activities are likely causing increased pain. But, if both light level and light MD show high correlations with pain, it is likely that pain is influenced by ambient light. In deployment 2,3, and 4, light features including light MD shown high correlation with pain frequency, suggesting that ambient light, not daytime activities, has effect on BTCP.

Interestingly, but perhaps not surprisingly, the correlation results showed wide variations across deployments. This suggests that patients and caregivers within a dyad react differently to their environmental surroundings. This highlights the importance of personalized cancer pain management strategies in the home setting, as what works for one patient may not work for another. By analyzing the environmental factors that are most strongly correlated with patient pain events, as reported by both patients and caregivers, healthcare providers can tailor interventions to the specific needs of each individual. For instance, if the broader correlation analyses indicate that a patient experiences more frequent pain episodes on days with greater exposure to high light levels, this information may be used to modify the home environment and the patient’s daily activities to reduce the light exposure.

### Future work

Future research should focus on further investigating the relationship between specific environmental features and BTCP in home settings. This may include utilizing additional advanced statistical methods such as in-depth feature selection techniques or deep learning models to uncover complex relationships and interactions between environmental factors and pain frequency/severity [42]. Advanced machine learning models and more data could be utilized to improve the, approximately, 70% pain event prediction accuracy. Other factors such as medication use, quality of life indicators, and psychological distress could also be examined for their impact on this relationship. For example, patients and caregivers self-reporting a lower mood or poor sleep may be more sensitive to their ambient surroundings, and subsequently experience pain differently. Interactions between environmental factors and the use of pain medications are also worth exploring, such as identifying specific environmental variables (e.g., noise reduction, lighting adjustments) that may influence the effectiveness of pain relief medications. As the BESI-C system evolves and deployments are scaled-up and more data are collected, our team plans to conduct these in-depth analyses.

### Limitations

One limitation is deciding which environmental data are most appropriate to use when the patient and caregiver are in separate rooms of their home when they each record a patient pain event. In these cases, it may be difficult to determine the role of specific environmental factors, such as light or noise, in the occurrence of pain. To address this, we utilized the user’s location information from the smartwatches and Bluetooth beacons to estimate patient and caregiver’s location. We have corrected this issue in subsequent iterations of the BESI-C system by adding a Ground Truth location question to our EMA schema (question: “what is your current location?” Responses: living room; bedroom; kitchen; outside the home; other) that participants answer when they record a pain event; this allows us to confirm location by both the EMA response and corresponding localization data. Another potential limitation of this pilot work is our number of deployments (*n* = 5) which may bias our correlation analysis results. However, it is important to note that from five deployments we collected a total of 283 user-initiated pain event EMAs (198 by patients; 85 by caregivers) and over 4200 hours of environmental data streams, which we argue is appropriate for a pilot study collecting real-world sensing data from critically ill patients and an important first step to explore a largely unexamined question - the influence of environmental factors on BTCP experiences within the home setting.

## Conclusions

Our research provides preliminary evidence of the potential impact of ambient environmental factors on BTCP in the home setting. We utilized real-world data to identify specific ambient environmental factors that may correlate with the frequency or severity of cancer pain, and conducted both individual and daily trend correlation analysis to provide a comprehensive understanding of the relationship between environmental factors and cancer pain.

Furthermore, our machine learning models showed promising results in predicting in-home BTCP from real-time environmental data streams. Our research suggests that personalized cancer pain management strategies in the home setting may benefit from a comprehensive understanding of the impact of individual and day-to-day ambient environmental factors on BTCP. By identifying specific environmental factors that correlate with cancer pain and utilizing machine learning models to predict in-home cancer pain from real-time environmental data streams, healthcare providers may be able to provide more effective and personalized pain management strategies for cancer patients.

### Supplementary Information

**Supplementary Material 1.**

## Data Availability

Not applicable.

## Abbreviations

BTCP: Breakthrough Cancer Pain
BESI-C: Behavioral and Environmental Sensing and Intervention system for Cancer
EMAs: Ecological Momentary Assessments
SD: Standard Deviation
MD: Median Deviation
MCR: Mean-Crossing-Rate
PT: Patient
CG: Caregiver
