# Supplementary material for: The influence of ambient environmental factors on breakthrough cancer pain: insights from remote health home monitoring and a proposed data analytic approach
Source: BMC Palliat Care. 2024 Mar 2;23:62. doi: 10.1186/s12904-024-01392-9 (PMC10908209; doi:10.1186/s12904-024-01392-9)
Supplement: Supplementary file 1 — Supplementary Material 1. [file 12904_2024_1392_MOESM1_ESM.docx]

**Appendix A: Performance Analysis of Ambient Environmental Time Window**

Here, the performance of predicting breakthrough cancer pain events using various time windows of environmental data prior to the patient’s pain event markers is shown. Different time window durations were also analyzed to determine the best performance as reported in the Figure A below. In figure A, a random forest model is trained on environmental data of different window sizes to predict upcoming BTCP. The results showed that using a 5-minute time window to predict upcoming BTCP events had an average MCC of 0.33 across five deployments, while a 15-minute time window had an MCC of 0.42, a 30-minute time window had an MCC of 0.38, and a 60-minute time window had an MCC of 0.28. Thus, the 15-minutes window of environmental data was used to analyze the individual pain episodes correlation and the predictive models for pain event prediction.


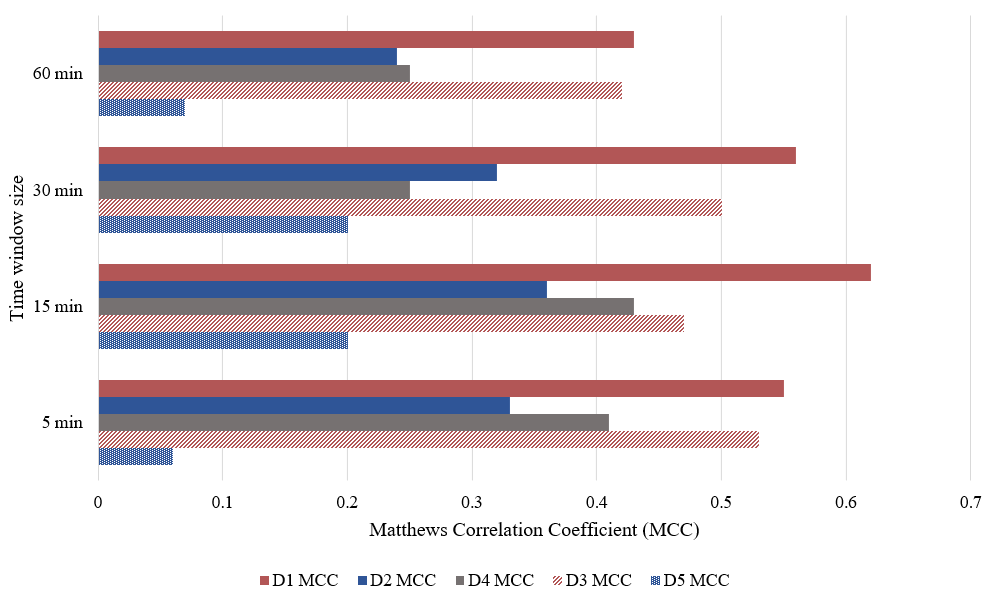


Figure A. Performance analysis of different environmental data windows and their impact on the random forest model’s MCC.

*Note: D = deployment*

**Appendix B: Machine Learning Models’ Hyperparameters**

The hyperparameters computed by the grid search method are as follow. We used the Gaussian Naïve Bayes model with 10^-9^ smoothing parameter. The DT used 5 maximum tree depth, 2 minimum samples required to split an internal node, and used maximum number of features considered when finding the best split equal to the square root of the number of features. The RF used 200 trees with max depth of 6 and minimum samples for split of 2. The SVM used polynomial kernel function, Regularization parameter of 0.1, and kernel coefficient (gamma) equal to $\frac{1}{number of features}$. The NN was set to have 1 hidden layers with 50 neurons, Rectified Linear Unit (ReLU) activation function, 0.01 initial learning rate with inverse scaling, ADAM solver for weight optimization, L2 regularization of 0.6, and 1,000 max number of epochs [34], [42].
